# Metformin causes cancer cell death through downregulation of p53-dependent differentiated embryo chondrocyte 1

**DOI:** 10.1186/s12929-018-0478-5

**Published:** 2018-11-15

**Authors:** Shu-Man Hsieh Li, Shu-Ting Liu, Yung-Lung Chang, Ching-Liang Ho, Shih-Ming Huang

**Affiliations:** 1 Department of Biochemistry, National Defense Medical Center, Taipei City, 114 Taiwan, Republic of China; 2 Division of Hematology/Oncology, Department of Medicine, Tri-Service General Hospital, National Defense Medical Center, Taipei City, 114 Taiwan, Republic of China

**Keywords:** Metformin, p53, Apoptosis, Reactive oxygen species, DEC1

## Abstract

**Background:**

Metformin is the most commonly used first-line medicine for type II diabetes mellitus. Acting via AMP-activated protein kinase, it has been used for more than 60 years and has an outstanding safety record. Metformin also offers protection against cancer, but its precise mechanisms remain unclear.

**Methods:**

We first examined the cytotoxic effects of metformin in the HeLa human cervical carcinoma and ZR-75-1 breast cancer cell lines using assays of cell viability, cleaved poly-ADP-ribose polymerase, and Annexin V-fluorescein isothiocyanate apoptosis, as well as flow cytometric analyses of the cell cycle profile and reactive oxygen species (ROS). We later clarified the effect of metformin on p53 protein stability using transient transfection and cycloheximide chase analyses.

**Results:**

We observed that metformin represses cell cycle progression, thereby inducing subG1 populations, and had induced apoptosis through downregulation of p53 protein and a target gene, differentiated embryo chondrocyte 1 (DEC1). In addition, metformin increased intracellular ROS levels, but *N*-acetyl cysteine, a ROS scavenger, failed to suppress metformin-induced apoptosis. Further results showed that metformin disrupted the electron transport chain and collapsed the mitochondrial membrane potential, which may be the cause of the elevated ROS levels. Examination of the mechanisms underlying metformin-induced HeLa cell death revealed that reduced stability of p53 in metformin-treated cells leads to decreases in DEC1 and induction of apoptosis.

**Conclusion:**

The involvement of DEC1 provides new insight into the positive or negative functional roles of p53 in the metformin-induced cytotoxicity in tumor cells.

## Background

The biguanide metformin is a first-line oral anti-hyperglycemic agent prescribed to nearly all newly diagnosed type II diabetes mellitus patients. Metformin also appears to have clinical benefits in other diseases, including diabetic nephropathy, stage III chronic kidney disease, and the cardiovascular complications associated with diabetes, such as cardiac hypertrophy and myocardial infarction [1, 2]. Although metformin has been used in Europe since 1957, the precise molecular mechanisms by which it mitigates diabetes are sill not fully understood. Results from an increasing number of studies suggest metformin works in the liver. Metformin enters liver cells primarily via organic cation transporter-1 and then suppresses mitochondrial complex I, which leads to a decrease in ATP levels and a rise in AMP levels [3]. The resultant change in the AMP/ATP ratio leads to activation of AMP-activated protein kinase (AMPK), which suppresses fatty acid synthesis and gluconeogenesis, and exerts insulin sensitizing effects [4].

Several studies indicate that metformin also exerts protective effects against inflammation, age-related pathologies, and cancer [2, 5–8]. Retrospective and prospective evidence suggests taking metformin is related to a decline in the incidences of various cancers, including hepatocellular carcinoma, pancreatic cancer, and colon cancer in diabetic patients [2, 7–10]. At present, there are at least 3860 articles listed in PubMed that are related to the study of metformin in cancer. Consequently, there are large amounts of both in vitro and in vivo data on the antitumor effects of metformin. Metformin is able to not only reduce insulin but also inhibit the mammalian target of rapamycin (mTOR) signaling pathway, which makes it an especially appealing target for evaluating metabolism unique to tumor cells, such as the Warburg effect [2, 9]. It has been proposed that the cancer preventive actions of metformin involve four components: 1) cancer stems cells, 2) microRNAs (miRs), 3) epithelial-to-mesenchymal transition (EMT), and 4) cellular senescence [9, 11, 12]. But as with diabetes, details of the mechanisms by which metformin acts against cancer remain unclear.

p53 is a well-known tumor suppressor gene that plays a vital role in about 50% of human cancers [13, 14]. At the premalignant tumor stage, senescence can be triggered normally by stressors such as DNA damage, oncogenes, and oxidative damage. In response to such stresses, p53 is activated and induces transcription of a variety of target genes to cause cells change their phenotypes from DNA repair to apoptosis and senescence [14, 15]. It appears, for example, that p53 transcriptional regulation of components of both the extrinsic (via a caspase cascade) and intrinsic (via the mitochondria) pathways serves as an apoptosis inducer [16, 17]. Moreover, p53 is also able to promote apoptosis through transcriptional induction of redox-related genes to generate reactive oxygen species (ROS), which in turn cause oxidative degradation of mitochondrial components and cell death [16, 18, 19]. Notably, metformin reduces the abundance of p53 in cases of hepatosteatosis, but enhances p53 stability via USP7 in esophageal cancer [20, 21]. Consequently, there is a need to confirm the availability and function of p53 in cancers treated with metformin in our working system. In the present study, therefore, we examined the cytotoxic effects of metformin in two cancer cell lines, clarifying its effects on apoptosis, ROS generation, mitochondrial function, differentiated embryo chondrocyte 1 (DEC1) expression, and p53 protein stability. Our findings provide new information for the potential reposition of metformin in the treatment of cancer.

## Methods

### Cell culture and reagents

HeLa cells were cultivated in Dulbecco’s modified Eagle’s medium (DMEM) supplemented with 10% fetal bovine serum (FBS) and 1% penicillin-streptomycin (Invitrogen, MA, USA). ZR-75-1 breast cancer cells were cultivated in Roswell Park Memorial Institute (RPMI) 1640 medium supplemented with 10% FBS and 1% penicillin-streptomycin (Invitrogen). Actinomycin D (Act D), cycloheximide (CHX), 2′,7-dichlorofluorescein diacetate (DCFH-DA), hydrogen peroxide (H_2_O_2_), metformin, MG132, *N*-acetyl cysteine (NAC), propidium iodide (PI), and thiazolyl blue tetrazlium bromide (MTT) were obtained from Sigma Aldrich (MO, USA). Pifithrin-α and Z-VAD-FMK was from Enzo (CA, USA).

### Cell survival analysis

Cells were seeded into 24-well culture plates and incubated for 1 day, after which they were exposed to different concentrations of metformin in fresh DMEM or RPMI 1640 for the indicated periods of time. After adding MTT solution (0.5 mg/ml in phosphate buffered saline, PBS) to each well, the cells were incubated for 1 h at 37 °C. Dimethylsulfoxide (DMSO; 200 μl) was then added, and the absorbance at 570 nm and 620 nm each was measured using an ELISA plate reader (Multiskan EX, Thermo, MA, USA). The control group containing cells cultured in medium only was defined as 100% cell survival.

### Western blotting

Cells were lysed at 4 °C in lysis buffer (100 mM Tris-HCl of pH 8.0, 150 mM NaCl, 0.1% SDS, and 1% Triton X-100). Proteins in the lysate were separated by SDS-PAGE, transferred onto polyvinylidene difluoride membranes (Millipore, MA, USA), and probed using antibodies against α-actinin (ACTN), cyclin D1, HSP90 α/β, p53(Santa Cruz Biotechnology, CA, USA), caspase 3, cyclin B1, EGFR, p-Histone H3 (phosphorylation at Ser 10, H3P), cleaved poly-ADP-ribose polymerase (cPARP) (Cell Signaling, MA, USA), and DEC1 (Bethyl Laboratory, TX, USA).

### Fluorescence-activated cell sorting (FACS), cell cycle profiles, apoptosis, and ROS analysis

Cells were fixed in 70% ice-cold ethanol and stored at − 30 °C overnight, after which they were washed with ice-cold PBS supplemented with 1% FBS twice and stained with PI solution (5 μg/ml PI in PBS, 0.5% Triton X-100, and 0.5 μg/ml RNase A) for 30 min at 37 °C in the dark. The cell cycle distribution was then evaluated based on cellular DNA content using FACS.

The incidence of apoptosis (early and late stages) and necrosis was assessed using a fluorescein isothiocyanate (FITC)-Annexin V Apoptosis Detection Kit (BD Biosciences, CA, USA) according to the manufacturer’s protocol.

The fluorescent marker DCFH-DA was used to determine intracellular ROS levels. Cells were incubated for 20 h with the indicated concentrations of metformin or with H_2_O_2_ as a positive control. Living cells were then stained with DCFH-DA (20 μM) for 40 min at 37 °C and harvested. After washed once with PBS, the cells were evaluated using a FACSCalibur flow cytometer and Cell Quest Pro software (BD Biosciences).

### Oxygen consumption rate (OCR)

The cellular OCR was detected using an XF24 bioenergetic assay according to the manufacturer’s protocol (Seahorse Bioscience, Billerica, MA, USA). In brief, HeLa cells were seeded onto an XF24 microplate in DMEM supplemented with 5% FBS. They were then incubated for 2 days, after which the medium was replaced with sodium bicarbonate-free DMEM supplemented with 1% FBS. The OCR was measured at a steady state, after which the machine sequentially added the standard samples (0.5 μM and 1 μM), oligomycin (1 μM), carbonyl cyanide 4-[trifluoromethoxy] phenylhydrazone (FCCP; 1 μM) and a mixture of rotenone (1 μM) and myxothiazol (1 μM) into the wells to obtain the maximal and non-mitochondrial respiration rates.

### Mitochondrial membrane potential analysis

Mitochondrial potential was measured using a BD™ MitoScreen Flow Cytometry Mitochondrial Membrane Potential Detection Kit (BD Biosciences) according to the manufacturer’s protocol. Briefly, HeLa cells were incubated for 18 h in a 6-cm culture plate and treated with metformin. The cells were then trypsinized and pelleted by centrifugation at 1000 rpm, after which the cells were resuspended in PBS and counted, which confirmed there were fewer than 1 × 10^6^ cells per ml. The cells were then stained with JC-1 dye (5,5′,6,6′-tetrachloro-1,1′,3,3′-tetraethylbenzimi-dazolylcarbocyanine iodide) for 10–15 min at 37 °C in a CO_2_ incubator. The fluorescence intensity of the JC-1 was evaluated flow cytometrically. The excitation wavelength was 488 nm, while emission wavelengths of 530 nm (FL1-H channel) and 580 nm (FL2-H channel) were used to detect the JC-1 monomer and aggregates, respectively.

### Plasmids and transfection

*DEC1* construct was produced by inserting the full-length polymerase chain reaction (PCR) product into pEGFP vector using the *Sac*I-*EcoR*I restriction sites. The pSG5.HA.p53 expression vector was constructed as described previously [22]. jetPEI (PolyPlus-transfection, France) reagent was used according to the manufacturer’s instructions to deliver the plasmids into cells cultivated in 6-well plates. The total amount of DNA in each well was adjusted to the same level by adding empty vector.

### Subcellular cytoplasmic, membrane, and nuclear extract preparations

HeLa cells were cultivated in 100-mm culture dishes and incubated under the indicated conditions. Cytoplasmic, membrane and nuclear extracts were separated using a Subcellular Protein Fractionation Kit for Cultured Cells according to the manufacturer’s protocol (Thermo Fisher Scientific, MA, USA). For the cytoplasmic fraction, cells were lysed in Cytoplasmic Extraction buffer (CEB) at 4 °C, after which the cytoplasmic extract (supernatant) was acquired by centrifugation (14,000 × *g*, 15 min, 4 °C). For the membrane fraction, CEB-treated pellets were lysed in Membrane Extraction buffer (MEB) at 4 °C, and the membrane extract (supernatant) was acquired by centrifugation (14,000 × *g*, 15 min, 4 °C). For the nuclear fraction, MEB-treated pellets were lysed in Nuclear Extraction buffer at 4 °C, and nuclear extract (supernatant) was acquired by centrifugation (14,000 × *g*, 15 min, 4 °C).

### Fluorescence microscopy

HeLa cells in 6-well culture plates were cultivated in DMEM supplemented with 10% FBS and 1% penicillin-streptomycin. After transfection with pEGFP.DEC1 for 5 h, a fluorescence microscope (Model DMURE2, Leica, Wetzlar, Germany) was used to observe cells expressing the encoded proteins, and Image-Pro®Plus software (Media-cybernetics, MD, USA) was utilized to process the images, as previously described [23].

### Reverse transcription-polymerase chain reaction (RT-PCR)

Total RNA was obtained using the TRIzol (Thermo Fisher Scientific) reagent according to the manufacturer’s protocol, after which 1 μg of the total RNA was reverse transcribed using MMLV reverse transcriptase (Epicentre Biotechnologies, WI, USA) for 60 min at 37 °C. A Veriti Thermal Cycler (Applied Biosystems, CA, USA) was utilized to run the PCR reactions. The PCR primers used were as follows: for GAPDH, 5’-CTTCATTGACCTCAACTAC-3′ (forward) and 5′-GCCATCCACAGTCTTCTG-3′ (reverse); for p53, 5′ -GATGAAGCTCCCAGAATGCCAGAG-3′ (reverse) and 5′ -GAGTTCCAAGGCCTCATTCAGCTC-3′ (reverse).

### *DEC-1* mRNA interference

*DEC-1-* and *LUC*-shRNA-containing lentiviral vectors were purchased from the National RNAi Core Facility (Academia Sinica, Taiwan, ROC). HeLa cells were infected with the indicated retroviruses or lentiviruses in selection medium containing 2 mg/ml polybrene. Forty-eight hours after infection, cells were treated with 8 mg/ml puromycin to select for a pool of puromycin-resistant clones. The silencing efficacy was verified by Western blot assay.

### Statistical analysis

Student’s t-test was used to compare the difference of apoptotic stages and cell viability by indicted agents. Values of *P* < 0.05 were considered significant.

## Results

### Metformin reduces cancer cell viability

**Fig. 1:**
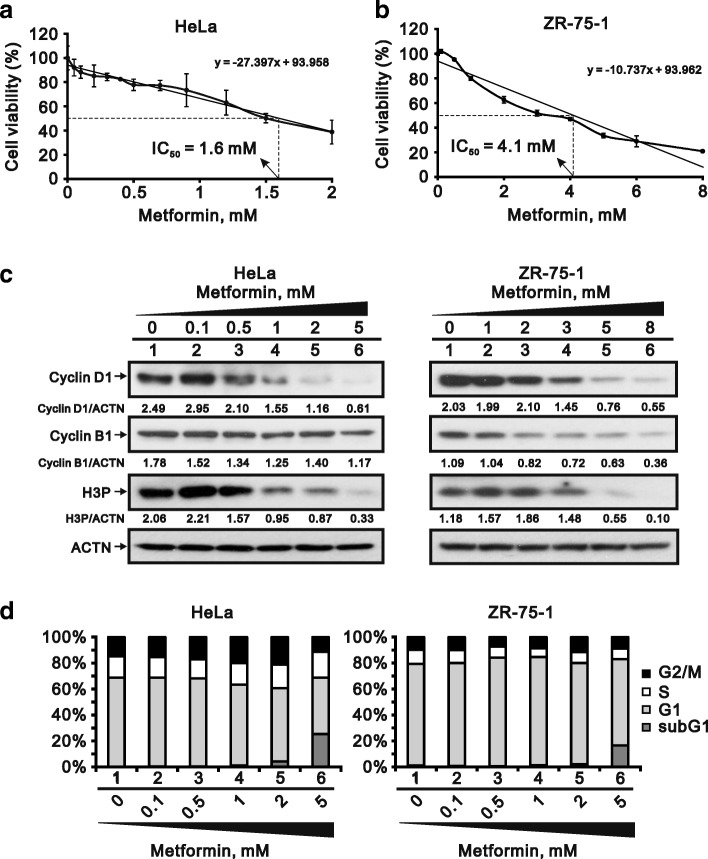
Cytotoxicity of metformin in HeLa and ZR-75-1 cells. **a** and **b** HeLa cells (**a**) and ZR-75-1 (**b**) cells were treated with the indicated concentration of metformin for 35 h and 70 h, respectively. Cell viability was measured using the MTT method. **c** HeLa and ZR-75-1 cells were incubated with indicated concentration of metformin for 20 h and 25 h, respectively. Cell lysates were subjected to western blot analysis using antibodies against cyclin D1, cyclin B1 and H3P. ACTN was the protein loading control. The protein levels of cyclin D1, cyclin B1 and H3P after normalization with the loading control protein ACTN are presented as fold change. **d** HeLa and ZR-75-1 cells were incubated with the indicated concentration of metformin for 30 h and 57 h, respectively. The cells were then subjected to flow cytometric cell cycle profile analysis. The results are representative of three independent experiments

We found that metformin reduced cell viability in the HeLa cervical carcinoma and ZR-75-1 breast cancer cell lines, exhibiting IC50s of 1.6 mM and 4.1 mM, respectively (Fig. 1a and b). We also observed that three proteins associated with cell cycle progression, cyclin D1 (G1 phase), cyclin B1 (G2 phase) and H3P (M phase), were dose-dependently suppressed by metformin (Fig. 1c). The cell cycle profiles in both cell lines showed that the subG1 population was dose-dependently increased by metformin while the G1 population was reduced (Fig. 1d). Thus cell cycle progression appears to be suppressed by metformin.

### Metformin induces apoptosis in HeLa cells

**Fig. 2:**
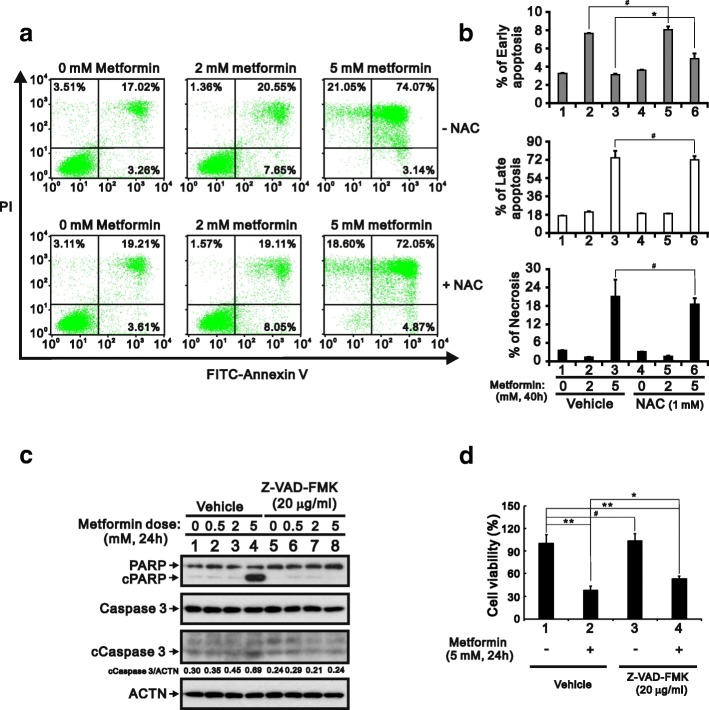
Metformin-induced apoptosis in HeLa cells. **a** HeLa cells (8 × 10^4^ cells) were incubated with the indicated concentration of metformin for 40 h, after which cell death, apoptosis or necrosis were quantified using FITC-Annexin V and PI and flow cytometry. **b** Quantitative analysis of percentage of indicated stages is presented as the mean ± S.D. of at least three independent experiments; # *p* > 0.05 and * *p* < 0.05 (Student’s t-test). **c** HeLa cells were incubated with the indicated concentration of metformin and 20 μg/ml Z-VAD-FMK for 24 h. Cell lysates were then subjected to western blotting with antibodies against PARP and caspase 3. ACTN was the loading control. The protein levels of cleaved Caspase 3 (cCaspase 3) after normalization with the loading control protein ACTN are presented as fold change. **d** HeLa cells (5 × 10^4^ cells) were treated with vehicle (DMSO), 5 mM metformin, 20 μg/ml Z-VAD-FMK, or 5 mM metformin plus 20 μg/ml Z-VAD-FMK for 24 h. Quantitative analysis of cell viability is presented as the mean ± S.D. of at least three independent experiments; # *p* > 0.05, * *p* < 0.05, and ** *p* < 0.01 (Student’s t-test)

Using FITC labeled Annexin V with PI in HeLa cells, we verified that metformin increases the incidence of apoptosis at both the early and late stages and necrosis (Fig. 2a and b). NAC failed to rescue the late apoptosis and necrosis, whereas it increased the percentage of early apoptosis. Consistent with that finding, western blot analysis of lysates from metformin-treated HeLa cell lysates revealed that levels of cPARP were increased in the metformin-treated cells (Fig. 2c). In addition, using the general caspase inhibitor Z-VAD-FMK, we found that metformin induced cleavage of PARP and caspase 3 and that approximately 40% of metformin-induced cell death was significantly rescued by Z-VAD-FMK (Fig. 2c and d). Taken together, these results suggest apoptosis may be a main cause of the cell death induced by metformin.

### Metformin promotes cytotoxic ROS generation unrelated to apoptosis

**Fig. 3:**
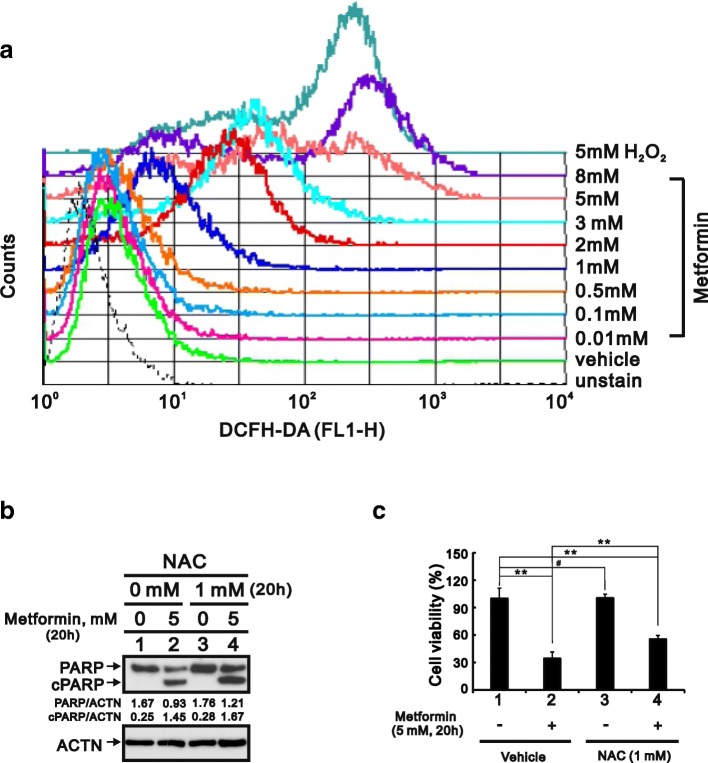
Induction of ROS generation by metformin in HeLa cells. **a** HeLa cells were incubated for 20 h with various concentrations of metformin, after which the live cells was stained with 10 μM DCFH-DA for 40 min at 37 °C and assayed using a flow cytometer. **b** HeLa cells were incubated for 20 h with 5 mM metformin and/or 1 mM NAC. The cell lysates were then subjected to western blotting with an antibody against PARP. ACTN was the loading control. The protein levels of PARP and cleaved PARP (cPARP) after normalization with the loading control protein ACTN are presented as fold change. **c** HeLa cells (5 × 10^4^ cells) were incubated for 20 h with vehicle (DMSO), 5 mM metformin, 1 mM NAC, or 5 mM metformin plus 1 mM NAC. Quantitative analysis of cell viability is presented as the mean ± S.D. of at least three independent experiments; # *p* > 0.05, * *p* < 0.05, and ** *p* < 0.01 (Student’s t-test)

We also used flow cytometry with DCFH-DA to examine the effect of metformin on intracellular ROS levels in HeLa cells. The results summarized in Fig. 3a show that with increases in the metformin concentration, ROS levels were obviously elevated relative the increases elicited by H_2_O_2_, which served as a positive control. Although ROS reportedly promote apoptosis [24], we discovered that the antioxidant NAC failed to prevent cPARP cleavage (Fig. 3b), but nevertheless partially suppressed metformin-induced cell death (Fig. 3c). Figs. 2 and 3 suggests ROS may contribute to metformin-induced cytotoxicity, though not via an apoptosis pathway.

**Fig. 4:**
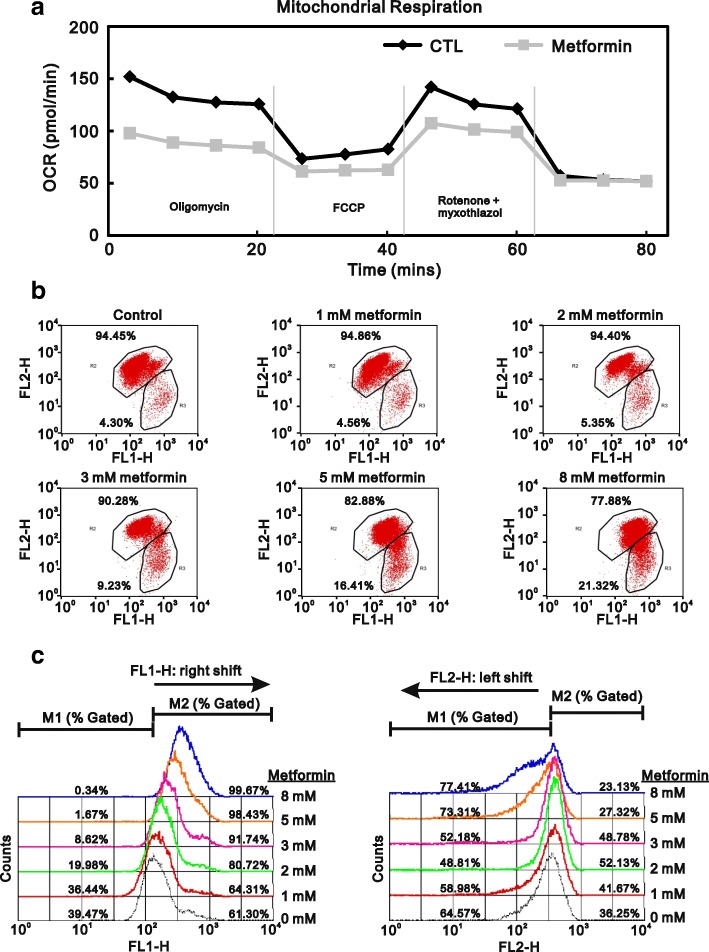
Effect of metformin on mitochondrial function in HeLa cells. **a** HeLa cells were incubated for 20 h with 5 mM metformin, after which the cellular OCR was measured in XF24 bioenergetic assays. **b** HeLa cells were incubated for 16 h with the indicated concentrations of metformin, after which JC-1 staining was analyzed using flow cytometry. **c** Changes in FL1-H and FL2-H were further evaluated using a FACS Calibur flow cytometer. The results are representative of two independent experiments

Alternatively, cellular ROS production could lead to disruption of mitochondrial respiration and/or collapse of the mitochondrial membrane potential [25, 26]. Metformin not only lowers blood glucose but also suppresses complex-I in the electric transport chain [27, 28]. For that reason, we measured the OCR and found that metformin decreased basal respiration, maximal respiratory capacity, and ATP-linked respiration in HeLa cells (Fig. 4a). To further examine the effect of metformin on mitochondrial membrane integrity, we used JC-1 staining to monitor the mitochondrial membrane potential. The results showed the metformin elicited increases in FL1-H (JC-1 monomers) and decreases in FL2-H (JC-1 aggregates) (Fig. 4b and c), which is indicative of depolarization of the mitochondrial membrane. This suggests metformin-induced ROS production in HeLa cells may reflect disruption of mitochondrial function.

### The functional role of DEC1 in metformin-induced apoptosis

**Fig. 5:**
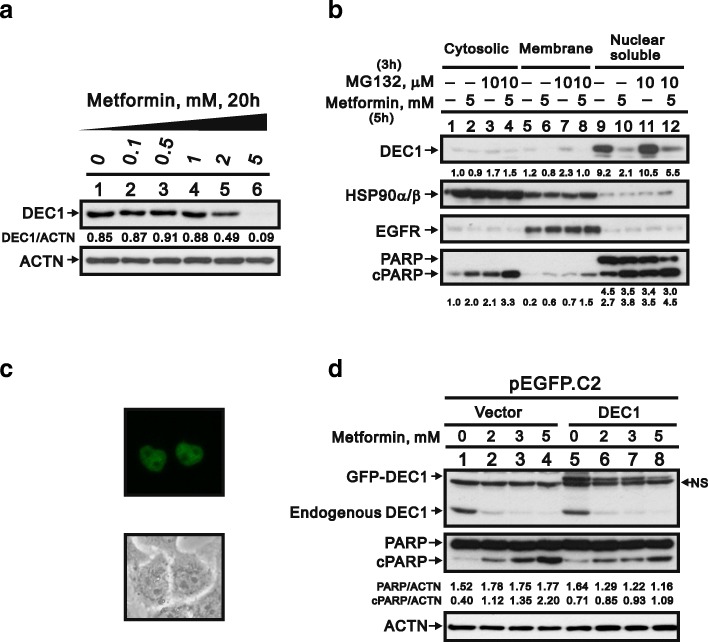
Function of DEC1 in metformin-induced apoptosis in HeLa cells. **a** HeLa cells were incubated for 20 h with the indicated concentrations of metformin, after which the cell lysates were subjected to western blotting with an antibody against DEC1. ACTN was the loading control. The protein levels of DEC1 after normalization with the loading control protein ACTN are presented as fold change. **b** HeLa cells were incubated with 5 mM metformin with and without 10 μM MG132 for the indicated times. They were then lysed; divided into cytoplasmic, membrane, and nuclear fractions; and subjected to western blotting with antibodies against DEC1, HSP90α/β (cytoplasmic fraction), EGFR (membrane fraction) and PARP (intact: nuclear fraction; cleaved: nuclear and cytoplasmic fractions). The protein levels of cleaved DEC1, PARP, and cPARP are presented as fold change. **c** HeLa cells were transiently transfected with 2 μg pEGFP.DEC1 for 5 h and then were observed with fluorescence-microscopy. **d** HeLa cells were transiently transfected with 2 μg of pEGFP vector and pEGFP.DEC1 and incubated for 13 h with 5 mM metformin. The cell lysates were subjected to western blotting with antibodies against DEC1 and PARP. ACTN was the loading control. The protein levels of PARP and cleaved PARP (cPARP) after normalization with the loading control protein ACTN are presented as fold change. The results are representative of three independent experiments

There is a link between the transcription factor DEC1 and apoptosis [29]. We observed that metformin reduced DEC1 levels and increased expression of cPARP proteins (Figs. 5a and 2b), which prompted us to evaluate DEC1’s involvement in metformin-induced apoptosis. We initially determined the localization of DEC1 by separately examining the cytoplasmic, membrane, and nuclear fractions of HeLa cells. HSP90α/β, EGFR and PARP served as markers of cytoplasm, cell membrane and nuclei, respectively. We found that DEC1 was present mainly in the nucleus, and that MG132, a proteasome inhibitor, had little or no effect of DEC1 levels. By contrast, metformin induced DEC1 degradation in all three fractions (Fig. 5b). Ectopic overexpression of pEGFP-DEC1 in HeLa cells confirmed that DEC1 localized primarily in the nucleus (Fig. 5c). To determine the relationship between DEC1 and apoptosis, HeLa cells were treated with metformin alone or in combination with ectopic DEC1 expression. Subsequent western blot analysis showed that ectopic DEC1 expression partially suppressed metformin-induced apoptosis (Fig. 5d), suggesting metformin may trigger apoptosis through downregulation of DEC1 expression.

### Involvement of p53 in metformin-induced apoptosis

**Fig. 6:**
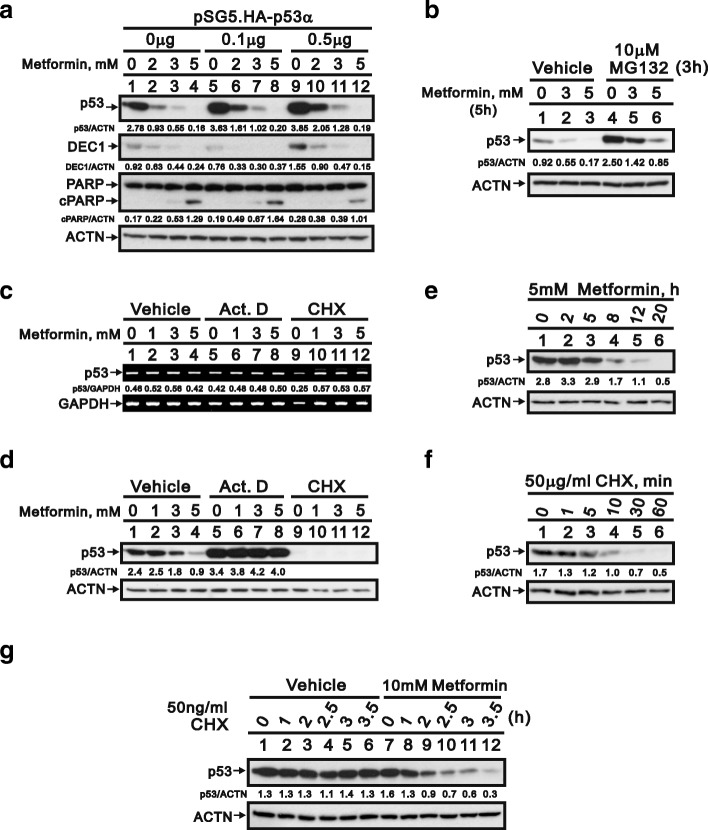
Transcriptional and translational regulation of p53 in HeLa cells. **a** HeLa cells were transiently transfected with 0.5 μg of pSG5.HA vector or the indicated amount of pSG5.HA.p53 and incubated for 12 h with 5 mM metformin. The cell lysates were subjected to western blotting with antibodies against p53, DEC1, and PARP. ACTN was the loading control. The protein levels of p53, DEC1, and cPARP after normalization with the loading control protein ACTN are presented as fold change. **b** HeLa cells were incubated for 5 h with the indicated concentrations of metformin with or without 10 μM MG132, after which the cell lysates were subjected to western blotting with an antibody against p53. ACTN was the loading control. The protein levels of p53 after normalization with the loading control protein ACTN are presented as fold change. **c** and **d** HeLa cells were incubated for 12 h with the indicated concentrations of metformin with and without 0.1 μM actinomycin D (Act D) or 50 μg/ml cycloheximide (CHX). Levels of p53 mRNA and protein were then assayed in the cell lysates using RT-PCR (**c**) and western blotting (**d**), respectively. GAPDH mRNA was the mRNA loading control; ACTN was the protein loading control. **e** and **f** HeLa cells were incubated with 5 mM metformin (**e**) or 50 μg/ml CHX (**f**) for the indicated times, after which cell lysates were subjected to western blotting with an antibody against p53. **g** HeLa cells were incubated for the indicated times with 10 mM metformin with and without 50 ng/ml CHX. The cell lysates were then subjected to western blotting with an antibody against p53. **d**-**g** The protein levels of p53 after normalization with the loading control protein ACTN are presented as fold change. The results are representative of three independent experiments

Early studies indicate *DEC1* gene is a target gene of p53 [30]. We found that metformin dose-dependently decreased levels of both p53 and DEC1 while making cells apoptotic. Overexpression of p53 partially rescued DEC1 levels and decreased the extent of apoptosis (Fig. 6a). These results suggest metformin may induce apoptosis in HeLa cells by acting on p53 upstream of DEC1. To better understand the mechanism underlying the downregulation of p53 by metformin, we first used MG132 to determine whether metformin induces degradation of p53 via a proteasome-dependent pathway. We observed that p53 degradation was mediated through the proteasomes, but MG132 failed to fully suppress p53 degradation elicited by metformin (Fig. 6b). Subsequent application of RNA and protein synthesis inhibitors (actinomycin D and cycloheximide, respectively) revealed no effect of metformin on p53 expression (Fig. 6c, compare lanes 1–4). Moreover, actinomycin D appeared to increased p53 levels and to exert a protective effect against metformin-induced p53 degradation (Fig. 6d, compare lanes 5–8).

Treatment with cycloheximide for 12 h elicited no further effect on p53 levels, most likely because p53 has a short half-life in HeLa cells (Fig. 6d, compare lanes 9–12) [31]. To overcome the time-window limitation for cycloheximide treatment, we re-examined the timing of metformin treatment and the stability of endogenous p53. Metformin-induced p53 degradation was first detected after around 2 h of treatment (Fig. 6e), but it was difficult to detect p53 in HeLa cells after only 10 min of cycloheximide treatment (50 μg/ml) (Fig. 6f), which is consistent with our earlier study [31]. We therefore decreased the cycloheximide concentration from 50 μg/ml to 50 ng/ml and increased the concentration of metformin from 5 to 10 mM. Under those conditions, metformin accelerated the degradation of p53 in the presence of cycloheximide. It thus appears that metformin reduces p53 levels in HeLa cells by reducing the protein’s stability (Fig. 6g).

### Loss-of-function of p53 and DEC1 for metformin-induced apoptosis

**Fig. 7:**
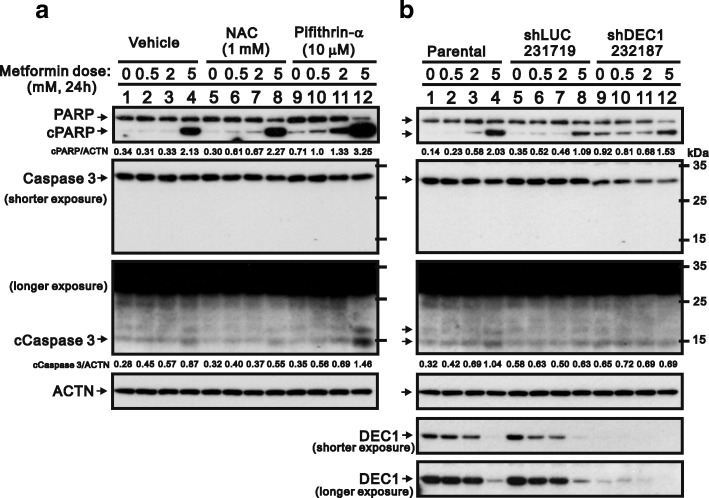
Verification of p53 and DEC1 into the metformin-induced apoptosis in HeLa cells. **a** HeLa cells were incubated for 24 h with the indicated concentrations of metformin, 1 mM NAC, and 10 μM pifithrin-α. The cell lysates were then subjected to western blotting with antibodies against PARP and caspase 3. ACTN was the loading control. **b** Following DEC1 knockdown in HeLa cells, the cells were incubated for 24 h with the indicated concentration of metformin. The cell lysates were then subjected to western blotting with antibodies against PARP and Caspase 3. ACTN was the loading control and DEC1 was the silencing efficiency of shDEC1. The protein levels of cPARP and cCaspase 3 after normalization with the loading control protein ACTN are presented as fold change. The results are representative of three independent experiments

To further verify the contribution of p53 and DEC1 to metformin-induced apoptosis, we applied a small-molecule inhibitor of p53, pifithrin-α, which reportedly inhibits several p53-dependent processes in vitro, including UV-induced expression of cyclin G, p21, and MDM-2 [32]. We also assessed the effect of DEC1 knockdown using a short-hairpin silencing system (Fig. 7). Our results showed that, by itself, pifithrin-α had no apparent effect on PARP cleavage. When combined 10 mM metformin, however, it dramatically increased levels of cPARP and caspase 3 (Fig. 7a). Knocking down DEC1 also increased levels of cPARP (Fig. 7b, compare lanes 9 with 7). In contrast to previously reported effects [33, 34], we observed that pifithrin-α induced apoptosis in HeLa cells, which is consistent with its promotion of p53-mediated apoptosis in JB6 cells [35].

## Discussion

**Fig. 8:**
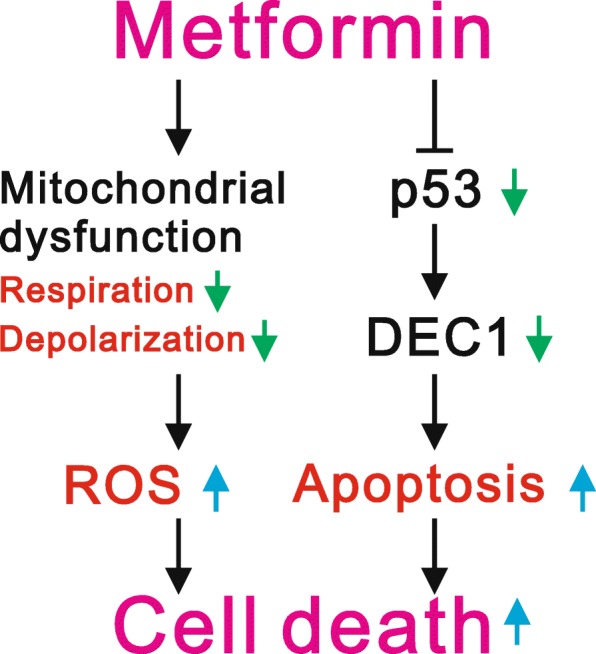
Proposed working mechanisms of metformin in cancer cells. Metformin may directly decrease p53 in sensitive cells, which would in turn downregulate expression of its target gene, *DEC1*, leading to apoptosis. Metformin not only induces cellular apoptosis but also induces ROS generation through repression of mitochondrial respiration and membrane potential to kill cancer cells. Thus, apoptosis, mitochondrial dysfunction, and ROS generation all contribute to the induction of HeLa cell death by metformin

Among the three biguanides (buformin, metformin. and phenformin) developed for the treatment of diabetes, increased cardiac mortality and risk of lactic acidosis led to withdrawal of buformin and phenformin in the early 1970s [2]. On the other hand, the lower risk and beneficial characteristics of metformin has enabled it to become one of the most popular medications in the world. Moreover, higher doses of biguanides may have direct antitumor effects [36]. Our findings suggest the antitumor effects of high-dose metformin are mediated through multiple pathways involving apoptosis, ROS generation, and downregulation of p53-related proteins. We propose that metformin may directly decrease p53 abundance in sensitive cells, which in turn leads to downregulation of p53 target genes (e.g., *DEC1*) and to induction of apoptosis. Metformin also induces ROS generation by suppressing the mitochondrial respiration rate and membrane potential. All of the abovementioned effects contribute to metformin-induced cell death (Fig. 8), and several clinical trials have shown promising results with metformin against various cancers, including breast cancer, gastric cancer, and pancreatic ductal adenocarcinoma, among others [37–39]. It is therefore crucial to further realize the molecular mechanisms of biguanides, which have the potential to contribute to the development in therapies for not only diabetes but also cancer.

Autophagy and apoptosis are two major cell death pathways [40]. One study showed that suppressing autophagy using chloroquine enhanced palmitic acid-induced apoptosis while increasing ROS generation [41]. However, our findings showed that NAC, a well-known ROS scavenger failed to inhibit the metformin-induced apoptosis and the cleavage of PARP. These results suggest that in our study apoptosis may be induced through suppression of autophagy, not through ROS generation. The precise relationship between autophagy and apoptosis remains to be further elucidated in HeLa cells. Several studies have demonstrated that metformin decreases ROS production through reduction of NAD (P) H oxidase activity [42, 43]. In addition to the inhibition of NAD (P) H oxidase activity, metformin may increase the ROS generation through other mechanisms in various cell types [44–46]. One study showed that there is complex interplay among processes regulating ROS, autophagy and apoptosis in response to expression of p53-inducible glycolysis and apoptosis regulator (TIGAR), which functions as a fructose 2,6-bisphosphatase [15]. TIGAR promotes activity in the pentose phosphate pathway and helps lower intracellular ROS. In the present study, metformin-mediated reduction in endogenous p53 may have led to ROS generation via downregulation of TIGAR expression.

The transcription factor p53 is a known tumor suppressor and is stabilized and activated for cellular responses to a variety of stresses, including hypoxia, DNA damage, and oncogene expression. These cellular responses include cell cycle arrest, apoptosis, senescence, and so on [13, 14]. It is commonplace for p53 activity and the activities of other tumor suppressors to be inhibited within tumors so as to inactivate cell-death pathways. Because HeLa cells are infected by human papillomavirus (HPV), they are able to express oncoprotein E6 to maintain endogenous wild-type p53 at low levels for human cervical tumorigenesis [22, 47]. Our findings in the present study suggest that as described in our earlier DXR work [48], metformin modulates levels of endogenous p53 to trigger various cellular activities altering expression of p53 target genes, including p21 (cell cycle inhibitor) [49], Bax (apoptosis inducer) [50], DNA damage regulated autophagy modulator 1 (autophagy inducer) [51], DEC1 (senescence inducer) [30], and survivin (apoptosis inhibitor) [52]. Given that p53 is an apoptosis inducer, the loss of survivin’s anti-apoptosis effect may explain why metformin, alone or in combination with pifithrin-α, induces apoptosis despite the reduction in p53 [52, 53]. However, further experiments will be needed to determine the contributions made by p53 and its target genes to the various dose-dependent effects of metformin. Several studies have shown that metformin may increase or decrease endogenous p53 via mechanisms involving miR-34a, USP7, and mitophagy [20, 21, 54]. Additional experiments will be required to clarify how metformin selectively mediates increases or decreases in the levels of p53.

It was recently reported that metformin reduces the risk of cervical cancer [55]. Although the mechanisms underlying the reduced risk remain to be explored, the authors of that study suggest the reduction in risk reflects metformin’s ability to reduce inflammation via inhibition of nuclear factor κB and STAT3 pathways. Taken together, that work and our present findings suggest metformin has the potential to suppress the proliferation and growth of cervical cancer cells and may be a useful addition to the currently used methods for the prevention and treatment of cervical cancer.

## Conclusions

In the present study, we demonstrated that metformin has several actions that suppress cell survival, including induction of apoptosis and ROS generation. The regulatory mechanisms of metformin may reflect its ability to reduce the stability, and thus the abundance, of endogenous wild-type p53. This would be expected to alter expression of many p53-dependent target genes responsible for various cell death pathways. Our work fully supports the reposition of metformin for cancer treatment or combination therapy with currently used cancer therapeutic agents.

## Abbreviations

Act D: Actinomycin D
ACTN: α-actinin
AMPK: AMP-activated protein kinase
CEB: cytoplasmic extraction buffer
CHX: cycloheximide
cPARP: cleaved poly-ADP-ribose polymerase
DCFH-DA: 2′,7-dichlorofluorescein diacetate
DEC1: Differentiated Embryo Chondrocyte 1
DMEM: Dulbecco’s modified Eagle’s medium
EMT: epithelial-to-mesenchymal transition
FACS: fluorescence- activated cell sorting
FBS: fetal bovine serum
FCCP: carbonyl cyanide 4-[trifluoromethoxy] phenylhydrazone
FITC: fluorescein isothiocyanate
H_2_O_2_: hydrogen peroxide
H3P: phosphorylation at Ser 10 Histone H3
JC-1: 5,5′,6,6′-tetrachloro-1,1′,3,3′-tetraethylbenzimi-dazolylcarbocyanine iodide
MEB: membrane extraction buffer
miRs: microRNAs
MTT: thiazolyl blue tetrazlium bromide
NAC: *N*-acetyl cysteine
NEB: nuclear extraction buffer
OCR: oxygen consumption rate
OCT1: organic cation transporter-1
HPV: human papillomavirus
PBS: phosphate buffered saline
PI: propidium iodide
ROS: reactive oxygen species
RPMI: Roswell Park Memorial Institute
RT-PCR: reverse transcription-polymerase chain reaction
TIGAR: TP53-inducible glycolysis and apoptosis regulator
